# Health Insurance as a Moderator of Cardiovascular Risk Among Adults with Depression: A Cross-Sectional and Geographic Analysis Using BRFSS 2019–2023 Data

**DOI:** 10.3390/healthcare14070843

**Published:** 2026-03-26

**Authors:** Amani Alharthy

**Affiliations:** Department of Health Science, College of Health and Rehabilitation Sciences, Princess Nourah bint Abdulrahman University, P.O. Box 84428, Riyadh 11671, Saudi Arabia; afalharthy@pnu.edu.sa

**Keywords:** depression, cardiovascular disease, insurance, United States, geographic disparity

## Abstract

**Background**: Depression is a key psychosocial risk factor for cardiovascular disease (CVD); however, the degree to which lack of health insurance amplifies this risk remains unclear. Geographical disparities in mental health, cardiovascular outcomes, and insurance coverage further complicate prevention efforts. Understanding the interplay between these factors is crucial for planning targeted interventions to mitigate population-level cardiovascular risk. **Objective**: To assess whether health insurance modifies the relationship between depression and CVD among adults and to illustrate geographic disparities in combined mental health, cardiovascular risk, and insurance burden across US states and territories. **Methods**: We analyzed Behavioral Risk Factor Surveillance System (BRFSS) data from 2019 to 2023, including adults aged ≥18 years with complete data on depression, CVD, and health insurance status (n = 457,670). Logistic regression models were employed to estimate the relationship between depression and CVD, adjusting for demographic and behavioral risk factors. An interaction term between depression and lack of health insurance was included to examine moderation. State-level prevalence estimates were used to construct a four-tier composite burden index incorporating depression, CVD, and rates of lack of health insurance. Choropleth maps were developed to depict geographic patterns. **Results**: Overall, 21.1% of adults were found to have depression, 12.3% had CVD, and 5.8% had no health insurance. Depression was significantly associated with CVD (adjusted OR = 1.69; 95% CI: 1.65–1.72). Lack of health insurance significantly moderated the relationship between depression and CVD (interaction OR = 1.33; 95% CI: 1.18–1.51, *p* < 0.001). Geographic mapping analyses demonstrated marked heterogeneity. Tier 4 (highest burden) states included Kentucky, West Virginia, Louisiana, Oklahoma, Texas, New Jersey, Guam, and the U.S. Virgin Islands. Many Tier 3 and Tier 4 regions were clustered in the South and Appalachia. **Conclusions**: Depression is strongly associated with an increased prevalence of CVD, and this association is further amplified among individuals without health insurance coverage. Geographic disparities demonstrate critical hotspots where simultaneous mental health challenges, elevated risk of CVD, and limited insurance converge, highlighting the need for combined, area-specific public health strategies and approaches addressing both mental and cardiovascular health.

## 1. Introduction

Cardiovascular disease (CVD) remains the leading cause of morbidity and mortality globally, with profound social and economic burdens [1,2,3]. Risk factors of CVD, such as hypertension, diabetes mellitus, overweight and obesity, and smoking status, have long been recognized in the development and progression of CVD [4,5]. However, growing evidence suggests that non-traditional psychosocial factors, specifically depression, play a substantial role in cardiovascular health [6]. Depression is related to behavioral and physiological pathways that increase the risk of CVD, including inflammation, autonomic dysfunction, and unhealthy lifestyle behaviors [7,8]. Despite this well-documented association, the relationship between depression and cardiovascular outcomes is complex, with access to healthcare services potentially mitigating these risks [9,10]. Health insurance, as a primary determining factor of healthcare access, may therefore serve as a protective mechanism, leading to early diagnosis, management of cardiovascular risk factors [11], and treatment of depression.

Access to healthcare services remains uneven across the United States [12,13], principally among individuals aged at least 18 years old, who are less likely than older adults to maintain consistent health insurance coverage. This population may experience delays in diagnosis and treatment of both depression and CVD, resulting in compounded health risks [14,15]. Geographic disparities in health insurance coverage further complicate this phenomenon, with differences by state exhibiting variations in Medicaid expansion, socioeconomic status, and healthcare infrastructure [16,17]. Understanding how these disparities interact with depression to influence CVD risk is essential for developing targeted public health strategies that prioritize both mental and cardiovascular health.

While previous studies have explored the associations between depression and CVD or between insurance coverage and general health outcomes, there is a scarcity of data on how health insurance modifies the relationship between depression and cardiovascular risk, particularly among young adults [6,11,18,19,20,21]. Furthermore, geographic analyses of these associations remain limited, despite the clear evidence of regional differences in both depression prevalence and access to healthcare services. This knowledge gap prevents the designing of targeted, geographic-specific strategies that could mitigate CVD risk among vulnerable populations.

The current study addresses these gaps in knowledge by investigating how health insurance can mitigate cardiovascular risk among adults with depression and how these effects differ across the geographic regions in the USA. By examining both individual-level and state-level patterns, it aims to contribute evidence that can guide interventions to improve access to mental health services, enhance cardiovascular prevention strategies, and reduce health inequities across the United States.

## 2. Materials and Methods

### 2.1. Study Design and Setting

This study employed a cross-sectional and geographic analysis by using data from the Behavioral Risk Factor Surveillance System (BRFSS) collected between 2019 and 2023. The BRFSS is a nationally representative, state-based survey of non-institutionalized U.S. adults, which gathers information on health behaviors, chronic conditions, and access to healthcare services.

### 2.2. Study Population

The study population included adults at least 18 years old, residing in different states of the USA, with complete data on depression, CVD, and health insurance status.

### 2.3. Data Source and Variables

Key variables in BRFSS included self-reported depression, specifically defined by the survey question asking if a respondent had ever been told by a doctor, nurse, or other health professional that they have a depressive disorder (including depression, major depression, dysthymia, or minor depression). This measure reflects a lifetime history of diagnosis rather than current symptomatology. CVD was similarly defined as a self-reported history of myocardial infarction, angina, or stroke. Health insurance status was captured as a binary variable (insured versus uninsured) based on the respondent’s status at the time of the interview. Specifically, participants were asked if they had any type of health care coverage, including health insurance, prepaid plans such as HMOs, or government plans such as Medicare or Indian Health Service. Consequently, our measure represents a point-in-time assessment of insurance status and does not capture longitudinal fluctuations or periods of uninsurance occurring earlier in the year. Additional covariates included in the analyses were sociodemographic and behavioral factors such as age (years), sex (male/female), body mass index (BMI in kg/m^2^), smoking status (Yes/No), exercise/physical activity (Yes/No), and physician-diagnosed diabetes, to control for potential confounding influences on the relationship between depression, insurance coverage, and CVD. Education was included as a proxy for socioeconomic status, which significantly influences health literacy and access to preventive care, while physical inactivity was included as a critical behavioral mediator known to independently influence both mental health and cardiovascular outcomes. The selection of these variables was guided by the established literature identifying them as key determinants of cardiovascular health and healthcare utilization patterns. BMI was used to categorize study population into underweight (<18.5), normal (18.5–24.9), overweight (25.0–29.9), and obese (≥30). Geographic identifiers (FIPS code) at the state level were used to conduct mapping analyses to examine regional patterns of depression, CVD, and insurance coverage at state levels.

### 2.4. Statistical Analysis

Descriptive statistics were initially computed to summarize study population characteristics including the prevalence of depression, CVD, and health insurance coverage. All categorical variables were presented as frequencies and percentages. Prevalence estimates of depression, CVD, and health insurance coverage were stratified by demographic subgroups, including sex and geographic region to identify patterns of variation across the population.

To assess the relationship between depression and CVD, logistic regression models were used. An initial unadjusted model (Model 1) estimated the crude association between depression and CVD without including any covariate in the model. Model 2 adjusted for potential confounders, including age, sex, education, BMI, smoking status, diabetes, and physical inactivity. Model 3 incorporated an interaction term between depression and health insurance status to assess whether insurance coverage modified the association between depression and CVD. Adjusted odds ratios (OR) with 95% confidence intervals (CIs) were estimated and reported for the strength of association and precision of estimates. *p*-value for the significance of interaction effects was evaluated to determine whether the association between depression and CVD varied by insurance status.

For geographic mapping analyses, state-level prevalence estimates of depression, CVD, and health insurance coverage were calculated. To depict geographic variation in population health risk, we computed a four-level composite burden index incorporating state-level prevalence of depression, CVD, and lack of health insurance coverage. Each indicator was categorized based on predefined epidemiologic thresholds derived from the national distribution and mean prevalence rates within the 2019–2023 BRFSS dataset. These thresholds were selected to represent meaningful gradients: Tier 1 reflects states performing better than the national average across all domains, while Tier 4 identifies states in the highest quartile of burden for at least one clinical or structural marker. This equal-weighting approach was chosen to reflect that mental health, clinical CVD, and structural insurance barriers are equally critical pillars of population health vulnerability. States were assigned to Tier 1 (low population burden) if all three parameters of depression, CVD, and insurance fell within the lowest ranges (depression ≤ 20%, CVD ≤ 12%, uninsured ≤ 5%). Tier 2 (moderate population burden) included states with a single domain in a middle range (depression 20.1 to 23%, CVD 12.1 to 14%, uninsured 5.1 to 7%). Tier 3 (elevated population burden) included states with two markers beyond the intermediate thresholds (depression 23.1 to 25%, CVD 14.1 to 16%, uninsured 7.1 to 9%). Lastly, Tier 4 (high population burden) was assigned to US states above the highest category in at least one indicator (depression > 25%, CVD > 16%, uninsured > 9%), indicating considerable clinical and structural vulnerability. This categorical scheme allowed us to synthesize multiple health domains into a unified metric for evaluating overall health burden across USA states and territories. Choropleth maps were created to visualize geographic patterns of four-level composite burden index incorporating state-level prevalence of depression, CVD, and lack of health insurance coverage. Analyses were conducted using R version 4.3.0 and SPSS version 26.

## 3. Results

### 3.1. Prevalence of Depression, CVD, and Health Insurance Coverage

The sample comprised 457,670 individuals. As shown in Table 1, overall prevalence of CVD, diabetes, and depression were 12.3%, 15.2%, and 21.1%, respectively, whereas almost everyone (94.2%) reported having some form of health insurance.

### 3.2. Sociodemographic Characteristics of Study Population

As shown in Table 1, about one-third (36.8%) of the study participants were less than 50 years old. With respect to gender distribution, 52.5% of study participants were female and 47.5% were male. More than two thirds (68.6%) reported having attended or graduated from college, and most study participants were from urban areas (86.5%). Lifestyle indicators revealed that 63.5% of the sample were non-smokers, and 76.5% had engaged in physical activity within the last one month. Based on BMI classifications, 36.6% were classified as overweight and 32.7% as obese.

### 3.3. Prevalence of Depression, Cardiovascular Disease, and Health Insurance Coverage by Demographic Subgroups and Geographic Regions

Table 2 exhibits the distributional characteristics of depression, CVD, and health insurance status across demographic and residential subgroups. Among the population with depression, the highest proportion was found in individuals under 50 years of age (44.9%) and among females (65.3%). In contrast, the CVD-positive cohort was characterized by an older demographic, with 54.7% of cases occurring among individuals aged 70 years or older, and a slightly higher representation of males (54.5%) than females (45.5%). Regarding healthcare access, the largest share of insured individuals (35.0%) fell within the 50 to 70-year age category. Across all three indicators, the vast majority of the burdened population resided in urban areas, accounting for 87.0% of those with depression, 83.3% of those with CVD, and 86.5% of those with health insurance coverage.

### 3.4. Model 1: Association Between Depression and CVD: Unadjusted Results

Findings from univariate logistic regression analyses (Model 1) revealed statistically significant associations (*p* < 0.001) between depression, other sociodemographic factors, and CVD (Table 3). The unadjusted logistic regression analysis revealed that depression was independently associated with 37% higher odds (OR = 1.37; 95% CI: 1.34 to 1.40) of CVD. With respect to other sociodemographic factors, age was the strongest predictor, with individuals aged ≥70 years revealing more than ten-fold increased odds of CVD compared to those aged <50 years (OR = 10.27; 95% CI: 9.95 to 10.59). Also, participants aged 50 to 70 years had more than five times the odds of CVD (OR = 5.06) compared to those aged <50 years. Among health conditions, diabetes was related to a three-fold increase in odds (OR = 3.28) of CVD, while smoking showed a two-fold increase (OR = 2.07) in odds of CVD compared to non-smokers. Educational level also demonstrated a significant gradient, with individuals who graduated high school having greater odds of CVD compared to those who did not (OR = 1.58) graduate high school. Obesity was the most predictive BMI group (OR = 1.39) for CVD compared to normal BMI. In contrast, physical activity appeared as the only protective determinant, associated with a significant reduction in odds (OR = 0.48) of CVD, indicating a more than 50% lower likelihood of CVD.

### 3.5. Model 2: Association Between Depression and CVD: Adjusted Results

In the multivariate logistic regression model (Model 2) adjusting for all covariates (Table 4), several demographic and health-related factors remained significantly associated with CVD (*p* < 0.001). Depression was independently associated with 69% higher odds (OR = 1.69; 95% CI: 1.65–1.72) of CVD after adding other factors (age, sex, education, geographic area, BMI, smoking, physical activity, and diabetes) in the model.

Age was found to be a strong predictor of CVD, with those aged ≥70 years demonstrating over nine-fold increased odds of CVD (OR = 9.54), and those aged 50 to 70 years establishing more than four-fold increased odds (OR = 4.39) of CVD compared to those <50 years. Sex was also a significant determinant of CVD, with males showing 60% higher odds (OR = 1.60) for CVD compared to females. Educational level demonstrated a graded association with CVD: graduating high school was associated with 42% higher odds (OR = 1.42) of CVD and attending or graduating college with 18% higher odds (OR = 1.18) of CVD, compared to not graduating from high school. Among behavioral and health factors, there was a 59% increase in odds (OR = 1.59) of CVD among smokers than non-smokers. BMI was positively associated with CVD, with odds ratios ranging from 1.07 for the overweight category to 1.17 for participants classified in the underweight group. Living in a rural vs. urban area was related to modestly increased odds (OR = 1.10) of CVD. Contrary to the other factors, exercise in the last 30 days was found to be the only protective factor of CVD, related to a 33% reduction in odds (OR = 0.67; 95% CI: 0.66 to 0.69) of CVD in the adjusted model (Table 4).

### 3.6. Model 3: Interaction Model to Assess Whether Insurance Coverage Modifies the Relationship Between Depression and CVD

In the final multivariate logistic regression model (Model 3 with the interaction between depression and health insurance), depression remained a strong independent determinant of CVD, related to more than double the odds of CVD (OR = 2.23; 95% CI: 2.17 to 2.29). Notably, the interaction between depression and lack of health insurance status was statistically significant (OR = 1.33; *p* = 0.001), revealing that depression had an amplified association among study participants with lack of health insurance coverage. Overall, age also remained the strongest predictor, with individuals aged ≥70 years showing more than nine-fold increased odds of CVD (OR = 9.27; 95% CI: 8.97 to 9.58), and those aged 50 to 70 years showing more than four-fold increased odds (OR = 4.31) compared to the individuals <50 years old. Diabetes (OR = 2.09), male sex (OR = 1.60), and smoking (OR = 1.59) were also significantly related to increased odds of CVD. Educational status demonstrated a graded association, with higher odds observed among those who graduated high school (OR = 1.42) or attended college (OR = 1.18), compared to those individuals who did not graduate from high school. In Model 3, BMI was also positively associated with CVD, ranging from OR = 1.07 for individuals who were overweight to OR = 1.17 for those in the underweight group. Residing in a rural region was associated with modestly increased odds (OR = 1.10), while physical activity was still found to be the only protective factor, associated with a 33% reduction in odds (OR = 0.67; 95% CI: 0.66 to 0.69) of CVD as shown in Table 5.

### 3.7. Mapping Analysis Results

#### 3.7.1. State-Level Prevalence Rates of Depression, CVD, and Insurance Coverage

Across US states and territories, the prevalence of depression averages 21.1%, with the highest prevalence of depression found in Kentucky (28.2%), West Virginia (27.1%), and Oregon (26.3%), while Hawaii (15.7%) and New Jersey (14.5%) reported the lowest prevalence of depression (Table 6). Overall, CVD affects about 12.3%, with West Virginia found to be the state with the highest prevalence (17.3%), alongside Kentucky (16.8%) and Arkansas (16.9%), whereas the District of Columbia (8.8%) and the U.S. Virgin Islands (7.5%) demonstrated the lowest prevalence of CVD. With respect to insurance coverage, overall national rate of uninsurance was 5.8%, with wide disparities across the USA: the U.S. Virgin Islands (15.3%), Texas (11.3%), and North Carolina (10.0%) have the highest proportions without insurance coverage, while D.C. (2.3%), Hawaii (2.5%), and Vermont (3.2%) showed near-universal coverage. Taken together, states in Appalachia and the South appear to reveal overlapping high rates of depression, CVD, and differences in insurance, emphasizing states and territories of clustered health vulnerability.

#### 3.7.2. Geographic Map Highlighting Regions with High Prevalence of Depression, Low Insurance Coverage, and Elevated CVD Risks

**Figure 1:**
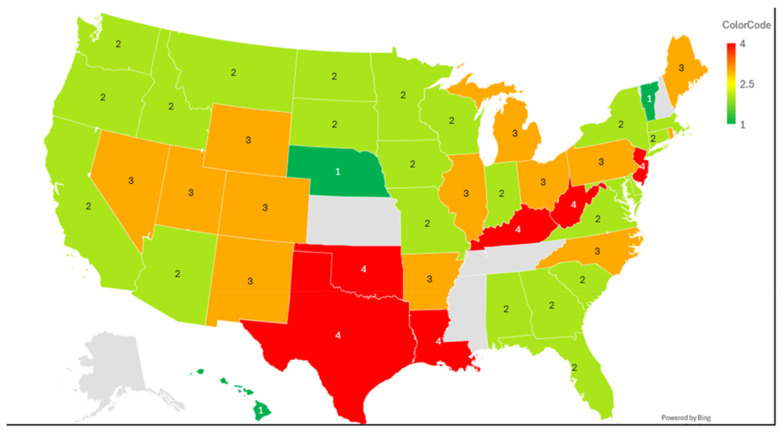
Composite health burden across US states: depression, CVD, and lack of insurance.

As shown below in the choropleth map, geographic grouping of the combined health burden showed substantial heterogeneity across the USA states and territories (Figure 1). Tier 4 regions, demonstrating the highest burden, were featured by substantially high depression and CVD prevalence and, in several cases, a high burden of uninsurance. Regions including Kentucky, West Virginia, Louisiana, and Oklahoma exhibited simultaneously high rates of depression and CVD, with Oklahoma additionally demonstrating an elevated burden of uninsurance. Texas revealed a unique profile characterized by the highest depression and a remarkably high burden of uninsurance, while New Jersey, Guam, and the U.S. Virgin Islands were classified in Tier 4 mainly owing to the high prevalence of uninsurance despite the relatively lower burden of two clinical indicators. Tier 3 encompassed states with elevations in two indicators, such as Arkansas, Ohio, Michigan, Maine, Rhode Island, Colorado, Illinois, Nevada, North Carolina, New Mexico, Pennsylvania, Utah, and Wyoming. Tier 2 included states with moderate concern, those displaying elevations in a single indicator, such as Alabama, Arizona, California, Connecticut, Delaware, Florida, Georgia, Idaho, Indiana, Iowa, Maryland, Massachusetts, Minnesota, Missouri, Montana, New York, North Dakota, Oregon, South Carolina, South Dakota, Virginia, Washington, Wisconsin, and Puerto Rico. Tier 1 states included the lowest overall burden of all three indicators, and this tier included Hawaii, the District of Columbia, and Vermont, as well as borderline low-burden states such as Georgia and Nebraska, which reached threshold values for one or more markers but were below the predefined threshold values.

## 4. Discussion

The current study provides evidence revealing that depression is strongly associated with CVD among US adults aged ≥18 years and the lack of health insurance is linked to a further elevation in this risk profile. Using nationally representative BRFSS data and a geographic burden index, the study revealed that not only individual-level risk patterns but also considerable state-level inequalities indicate wider structural inequities in the USA’s healthcare system. These results provide new insights into the psychosocial and healthcare access aspects of cardiovascular risk and emphasize the significance of policies that incorporate mental health and preventive cardiovascular care.

Consistent with the existing literature, depression in the current study appeared to be a significant independent predictor of CVD after controlling key demographic and behavioral covariates in the model. This relationship between depression and CVD aligns with the prevailing literature connecting depression to physiologic processes, including but not limited to chronic inflammation, dysregulation of the hypothalamic–pituitary–adrenal axis, endothelial dysfunction, and increased sympathetic activity [14,22,23]. Depression is also associated with behavioral risk factors such as lack of exercise, smoking, and poor dietary intake, which may complicate physiological mechanisms and elevate cardiovascular injury [24,25].

A distinct contribution of the current study is that the depression–CVD relationship is moderated by lack of health insurance. The significant interaction term reveals that uninsured individuals with depression exhibit a disproportionately higher likelihood of CVD compared to their insured counterparts, even after adjusting for clinical and demographic factors. These findings imply that access to healthcare has a buffering effect, probably via earlier recognition and management of both mental health conditions and cardiovascular risk factors [26,27]. Health insurance coverage enhances access to medications such as antidepressants, psychotherapy, physician services, primary health care, lipid control, blood pressure management, and preventive screening—all of these can reduce the progression of CVD [26,28]. Conversely, uninsured individuals may not seek timely care until symptoms become severe, leading to co-occurrence of poorly managed depression and advanced cardiovascular conditions.

The geographic element of the current study provides supplementary insights into how structural determinants drive health disparities. Rather than merely identifying high-prevalence areas, the tiered index highlights meaningful regional clustering of health vulnerabilities that reflect systemic inequities. Tier 4 states, primarily clustered in the South and Appalachia, represent a “syndemic” of risk, where chronic poverty and inadequate mental health infrastructure converge with lifestyle-related cardiovascular risks. In states like Kentucky and West Virginia, the high shared burden suggests that clinical interventions alone may be insufficient without addressing the underlying socioeconomic constraints and the shortage of behavioral health providers.

The inclusion of Texas, New Jersey, and US territories in Tier 4—driven primarily by uninsurance—shifts the interpretation from individual pathology to structural policy. This emphasizes a prominent phenomenon: high rates of uninsurance serve as a catalyst for population vulnerability by restricting access to the very preventive services that could break the link between mental distress and physical decline [29]. In Texas, the absence of Medicaid expansion likely perpetuates a cycle of unmanaged chronic conditions, while in territories like Guam, geographic isolation further compounds these structural barriers [30]. These patterns demonstrate that population-level cardiovascular risk is shaped more by state-level policy decisions and infrastructure than by individual health behaviors alone.

The variation observed across Tiers 1 through 3 suggests a “policy-health gradient”. Tier 1 states, such as Hawaii and Vermont, illustrate the potential success of proactive healthcare modeling, where longstanding commitments to insurance mandates and primary care investment correlate with lower clinical burdens. Conversely, the “intensifying pressures” seen in Tier 3 states may signal transitional phases where recent shifts in resource allocation or insurance coverage have yet to yield population-level improvements. Together, these findings emphasize that the interplay of depression and CVD is a complex byproduct of healthcare policy and socioeconomic circumstances, requiring targeted, area-specific structural reforms rather than a one-size-fits-all approach.

Together, these geographic findings emphasize that the interplay between depression, CVD, and insurance coverage is not uniformly distributed across the USA. Rather, it exhibits a complex interaction of healthcare policy, socioeconomic circumstances, population health behaviors, and availability of mental health services. Territories with the maximum burden often experience constant and sustainable structural discrimination including limited Medicaid expansion, rural health challenges, shortages in healthcare workforce, and financial constraints that delay access to both mental health and cardiovascular care.

## 5. Strengths and Limitations

The current study has numerous key strengths. First, it leverages nationally representative BRFSS data of five years (2019 to 2023), with a large sample size that ensures stable and precise prevalence estimates across demographic subgroups and geographic areas. The use of a standardized, state-based surveillance system improves comparisons across various states and territories, improving the reliability of both individual-level and geographic results. Second, by combining depression, CVD, and coverage of health insurance into a complex four-tier burden index, the analysis exceeds beyond traditional single-metric assessments and provides a more comprehensive view of interconnecting clinical and structural susceptibilities. This multidimensional lens offers a better understanding of where population health risks cluster and recognizes geographic areas that require important attention. Third, the study includes a moderation analysis that assessed whether insurance coverage modifies the relationship between depression and CVD, providing insights into an underexplored area of interest. Finally, integrating statistical modeling with geographic mapping provides a more actionable evidence base for policymakers, allowing targeted allocation of resources and state-specific approaches to improve care. However, the potential for residual confounding remains. While the study adjusted for key factors such as age, education, and diabetes, other significant confounders—including household income, specific comorbid chronic conditions beyond diabetes, and actual frequency of healthcare utilization—were either unavailable or not included in the primary models. These unmeasured factors may influence both insurance status and cardiovascular outcomes, potentially impacting the observed associations.

However, numerous limitations should be acknowledged. The cross-sectional design precludes causal inference, limiting our ability to establish the temporal relationship between depression, lack of insurance, and CVD. Consequently, we cannot determine whether the lack of insurance preceded the development of depression or cardiovascular outcomes. Furthermore, the use of pooled data from 2019 to 2023 aimed to provide sufficient statistical power for stable state-level geographic mapping. However, this approach treats the five-year period as a single cross-section, which may obscure temporal trends or the impact of specific health insurance policy shifts (e.g., Medicaid expansion or federal subsidies) that occurred during this timeframe. Consequently, the findings represent an aggregate assessment of the depression–CVD–insurance relationship over the post-2019 period.

All parameters, including depression and CVD, were self-reported, which may result in a potential recall and social desirability bias. Furthermore, these measures rely on clinical diagnosis, which introduces the risk of misclassification bias. Specifically, differential reporting may occur by insurance status; individuals with health insurance have greater access to clinical screenings and are therefore more likely to receive a formal diagnosis of depression or CVD compared to uninsured individuals. This “detection bias” may result in an underestimation of the true disease burden among the uninsured population. Similarly, geographic regions with healthcare provider shortages (e.g., rural Appalachia) may exhibit lower reported prevalence due to limited diagnostic opportunities rather than lower actual disease incidence. The BRFSS excludes institutionalized adults, such as those in long-term care or correctional facilities, who may be at higher risk of depression and CVD, possibly underestimating the true prevalence of these conditions.

Insurance coverage was also considered a dichotomous variable, restricting nuanced interpretation of underinsurance, continuity of health insurance coverage, and quality of chosen plan. Furthermore, because insurance is measured at the time of the survey, the data may not reflect a participant’s cumulative lifetime exposure to being uninsured. This is particularly relevant for adults aged 65 and older; because Medicare coverage is near-universal in this demographic, an individual who spent decades uninsured may currently be classified as “insured”. Consequently, our findings may underestimate the “weathering” effect of long-term uninsurance on cardiovascular health.

In addition, the reliance on binary indicators for depression and health behaviors (e.g., smoking) limits our ability to account for disease severity, duration, or chronicity. The “ever diagnosed” metric for depression groups together a wide variety of clinical experiences, from a single remote episode to a prolonged, multi-year struggle. Given that the physiological mechanisms linking depression to CVD, such as chronic inflammation and autonomic dysfunction, are often dose-dependent or linked to the chronicity of mental distress, this lack of granularity likely provides a conservative estimate of the true associations. However, these standardized measures are essential for maintaining the large sample sizes required for state-level geographic analysis and represent the most reliable population-level data available for US-wide surveillance. Finally, while the four-tier composite burden index provides a novel synthesis of regional risk, the classification of states is sensitive to the specific categorical thresholds employed. Although our thresholds were based on the observed data distribution to ensure regional differentiation, alternative cut-off points might shift the classification of borderline states. Future research should explore weighted indices to further refine the prioritization of geographic hotspots.

## 6. Policy Implications

From a policy perspective, the study’s findings strengthen the importance of increasing insurance coverage, combining mental and cardiovascular health services, and financing preventive care. Specifically, our results suggest that expanding Medicaid in Tier 4 states, particularly those where uninsurance is a primary driver of risk, could serve as a structural intervention to decouple the link between depression and CVD. Practically, healthcare systems in high-burden geographic hotspots should prioritize “integrated care models” where cardiovascular risk screening is a standard component of mental health treatment, and conversely, depression screening is mandated in cardiac rehabilitation settings.

Furthermore, the geographic heterogeneity identified in this study supports the transition from broad national initiatives to area-specific public health strategies. For example, in rural Appalachia (Tier 4), practical approaches should include the expansion of telemedicine for mental health to overcome provider shortages, whereas in urban centers with high uninsurance, the focus should remain on subsidized coverage and mobile cardiovascular screening clinics. By aligning clinical interventions with the specific structural deficits of each region, policymakers can more effectively mitigate the combined burden of mental and cardiovascular health inequities.

## 7. Conclusions

In summary, the current study found that depression was independently associated with CVD and that the lack of health insurance further amplifies this association. Addressing both mental health and insurance coverage concurrently signifies a strategic opportunity to potentially decrease the burden of CVD and improve health equity across the different regions of the USA.

## Figures and Tables

**Table 1:** Sociodemographic and clinical characteristics of the study participants (n = 457,670).

Variable	Category	Frequency (n)	Percent (%)
Age Groups	<50	168,305	36.80%
	50 to 70	157,722	34.50%
	≥70	131,643	28.80%
Sex	Male	217,507	47.50%
	Female	240,163	52.50%
Education Level	Did not graduate HS	27,289	6.00%
	Graduated HS	116,478	25.50%
	Attended college or college graduate	313,903	68.60%
Geographic Area	Urban	395,930	86.50%
	Rural	61,740	13.50%
Smoking	No	290,428	63.5%
	Yes	167,242	36.5%
Exercise	No	107,609	23.5%
	Yes	350,061	76.5%
BMI (kg/m^2^)	Underweight (<18.5)	7977	1.7%
	Normal (18.5–24.9)	132,310	28.9%
	Overweight (25.0–29.9)	167,686	36.6%
	Obese (≥30)	149,697	32.7%
Diabetes	No	388,311	84.80%
	Yes	69,359	15.20%
Depression	No	361,032	78.90%
	Yes	96,638	21.10%
CVD	No	401,328	87.70%
	Yes	56,342	12.30%
Any Health Insurance	No	26,448	5.80%
	Yes	431,222	94.20%

CVD: Cardiovascular disease, BMI: Body mass index, HS: High School.

**Table 2:** Demographic and geographic composition of individuals with depression, cardiovascular disease (CVD), and lack of health insurance.

Subgroup	Category	Depression (n, %)	CVD (n, %)	Health Insurance (n, %)
Age	<50 years	43,395 (44.9%)	4866 (8.6%)	149,884 (34.8%)
	50–70 years	34,292 (35.5%)	20,660 (36.7%)	151,031 (35.0%)
	≥70 years	18,951 (19.6%)	30,816 (54.7%)	130,307 (30.2%)
Sex	Male	33,497 (34.7%)	30,696 (54.5%)	202,638 (47.0%)
	Female	63,141 (65.3%)	25,646 (45.5%)	228,584 (53.0%)
Geographic region	Urban	84,045 (87.0%)	46,926 (83.3%)	373,025 (86.5%)
	Rural	12,593 (13.0%)	9416 (16.7%)	58,197 (13.5%)

**Table 3:** Association between depression and other factors with CVD: unadjusted results.

Variable	Category/Level	OR	95% CI	*p*-Value
Depression	Yes (vs. No)	1.37	1.34–1.40	<0.001
Age Groups	50 to 70 years (vs. <50 years)	5.06	4.90–5.23	<0.001
	≥70 years (vs. <50 years)	10.27	9.95–10.59	<0.001
Sex	Male vs. Female	1.37	1.35–1.40	<0.001
Education Attainment	Graduated HS (vs. Did not graduate HS)	1.58	1.52–1.63	<0.001
	College/Graduate (vs. Did not graduate HS)	1.27	1.25–1.30	<0.001
Geographic Area	Rural (vs. Urban)	1.34	1.31–1.37	<0.001
Smoking	Yes (vs. No)	2.07	2.03–2.10	<0.001
Physical Activity	Yes (vs. No)	0.48	0.48–0.49	<0.001
BMI	Underweight (<18.5) (vs. Normal)	1.24	1.16–1.33	<0.001
	Overweight (25.0 to 29.9) (vs. Normal)	1.25	1.22–1.28	<0.001
	Obese (≥30) (vs. Normal)	1.39	1.36–1.42	<0.001
Diabetes	Yes (vs. No)	3.28	3.22–3.35	<0.001

CVD: Cardiovascular disease, BMI: Body mass index, HS: High School.

**Table 4:** Association between depression and CVD: adjusted results.

Variable	Category/Level	AOR	95% CI	*p*-Value
Depression	Yes (vs. No)	1.69	1.65–1.72	<0.001
Age Groups	50–70 years (vs. <50 years)	4.39	4.25–4.54	<0.001
	≥70 years (vs. <50 years)	9.54	9.24–9.86	<0.001
Sex	Male vs. Female	1.6	1.57–1.63	<0.001
Education Level	Graduated HS (vs. Did not graduate HS)	1.42	1.37–1.48	<0.001
	College/Graduate (vs. Did not graduate HS)	1.18	1.16–1.21	<0.001
BMI	Underweight (<18.5) (vs. Normal)	1.17	1.08–1.25	<0.001
	Overweight (25.0–29.9) (vs. Normal)	1.07	1.05–1.10	<0.001
	Obese (≥30) (vs. Normal)	1.15	1.12–1.18	<0.001
Geographic Area	Rural (vs. Urban)	1.10	1.07–1.12	<0.001
Smoking	Yes (vs. No)	1.59	1.56–1.62	<0.001
Diabetes	Yes (vs. No)	2.10	2.05–2.14	<0.001
Physical Activity	Yes (vs. No)	0.67	0.66–0.69	<0.001

CVD: Cardiovascular disease, BMI: Body mass index, HS: High School.

**Table 5:** Model 3: Interaction model to assess whether insurance coverage modifies the relationship between depression and CVD.

Variable	Category/Level	OR	95% CI	*p*-Value
Depression	Yes (vs. No)	2.23	1.97–2.51	<0.001
Health Insurance	No vs. Yes	1.46	1.36–1.56	<0.001
Interaction Term	Depression*Lack of Insurance	1.33	1.18–1.51	<0.001
Age Groups	≥70 years (vs. <50 years)	9.27	8.97–9.58	<0.001
	50–70 years (vs. <50 years)	4.31	4.17–4.45	<0.001
Sex	Male vs. Female	1.6	1.57–1.64	<0.001
Education Level	Graduated High School (vs. Did not graduate HS)	1.47	1.42–1.53	<0.001
	College/Graduate (vs. Did not graduate HS)	1.19	1.16–1.21	<0.001
Geographic Area	Rural (vs. Urban)	1.09	1.07–1.12	<0.001
Physical Activity	Yes (vs. No)	0.67	0.66–0.69	<0.001
BMI	Obese (≥30) (vs. Normal)	1.15	1.12–1.17	<0.001
	Underweight (<18.5) (vs. Normal)	1.17	1.09–1.26	<0.001
	Overweight (25.0–29.9) (vs. Normal)	1.07	1.04–1.09	<0.001
Smoking	Yes (vs. No)	1.59	1.56–1.62	<0.001
Diabetes	Yes (vs. No)	2.09	2.05–2.14	<0.001

CVD: Cardiovascular disease, BMI: Body mass index, HS: High School; The reference category for insurance status is “Has Health Insurance”. The reference category for depression is “No Depression”. The interaction term (No Insurance*Depression) represents the odds of CVD among individuals concurrently experiencing both conditions relative to the baseline reference groups.

**Table 6:** State-level prevalence rates of depression, CVD, and insurance coverage.

State/Territory	FIPS Code	%Depression	%CVD	%Without Insurance
Alabama	1	23.5%	15.6%	5.6%
Alaska	2	20.3%	11.1%	5.7%
Arizona	4	19.4%	12.8%	6.4%
Arkansas	5	24.1%	16.9%	6.0%
California	6	19.3%	11.2%	6.1%
Colorado	8	22.6%	9.6%	8.3%
Connecticut	9	19.3%	10.2%	5.4%
Delaware	10	19.5%	15.3%	5.1%
District of Columbia	11	19.7%	8.8%	2.3%
Florida	12	18.6%	14.8%	7.0%
Georgia	13	18.0%	13.8%	7.2%
Hawaii	15	15.7%	11.6%	2.5%
Idaho	16	21.0%	9.7%	8.5%
Illinois	17	21.0%	10.3%	8.9%
Indiana	18	23.2%	13.5%	5.5%
Iowa	19	18.2%	11.4%	5.8%
Kentucky	21	28.2%	16.8%	5.3%
Louisiana	22	25.9%	15.1%	4.6%
Maine	23	24.7%	13.9%	4.0%
Maryland	24	19.4%	12.1%	5.8%
Massachusetts	25	21.1%	9.7%	3.3%
Michigan	26	25.3%	14.2%	3.5%
Minnesota	27	23.1%	10.9%	5.5%
Mississippi	28	20.0%	11.9%	6.3%
Missouri	29	22.4%	13.5%	5.2%
Montana	30	21.5%	12.5%	5.4%
Nebraska	31	16.6%	12.7%	7.2%
Nevada	32	20.8%	13.7%	8.2%
New Jersey	34	14.5%	9.6%	9.7%
New Mexico	35	19.5%	11.3%	7.4%
New York	36	18.3%	10.7%	4.9%
North Carolina	37	22.5%	10.4%	10.0%
North Dakota	38	18.8%	12.9%	3.7%
Ohio	39	25.0%	15.2%	3.7%
Oklahoma	40	25.4%	14.7%	7.8%
Oregon	41	26.3%	11.6%	4.9%
Pennsylvania	42	23.1%	12.3%	7.4%
Rhode Island	44	25.2%	11.6%	4.4%
South Carolina	45	19.2%	15.1%	5.6%
South Dakota	46	18.0%	12.8%	4.4%
Texas	48	22.9%	11.3%	11.3%
Utah	49	22.3%	9.6%	7.4%
Vermont	50	25.1%	11.4%	3.2%
Virginia	51	20.1%	14.2%	3.6%
Washington	53	23.8%	10.5%	5.1%
West Virginia	54	27.1%	17.3%	4.5%
Wisconsin	55	21.1%	15.0%	4.7%
Wyoming	56	20.6%	13.3%	7.1%
Guam (Territory)	66	12.6%	12.5%	9.8%
Puerto Rico (Territory)	72	19.0%	10.3%	4.3%
U.S. Virgin Islands (Territory)	78	8.5%	7.5%	15.3%

## Data Availability

The data used in this study are publicly available from the Behavioral Risk Factor Surveillance System (BRFSS) database provided by the Centers for Disease Control and Prevention (CDC). The datasets analyzed during the current study are available at https://www.cdc.gov/brfss/ (accessed on 10 September 2025).

## Abbreviations

The following abbreviations are used in this manuscript:

CVD	Cardiovascular diseases
BRFSS	Behavioral Risk Factor Surveillance System
CIs	Confidence Intervals
